# Maternal Mental Health and Parenting Stress and Their Relationships to Characteristics of the Child With Fragile X Syndrome

**DOI:** 10.3389/fpsyt.2021.716585

**Published:** 2021-11-24

**Authors:** Lauren Bullard, Danielle Harvey, Leonard Abbeduto

**Affiliations:** ^1^MIND Institute, UC Davis Health, Sacramento, CA, United States; ^2^Department of Psychiatry and Behavioral Sciences, UC Davis Health, Sacramento, CA, United States; ^3^Division of Biostatistics, Department of Public Health Sciences, University of California, Davis, Davis, CA, United States

**Keywords:** *FMR1* carriers, fragile X syndrome, mental health, parenting stress, telehealth

## Abstract

Although previous research supports the notion that characteristics of both the child and the mother impact maternal well-being and parenting stress in mothers of children with FXS, more work is needed in which self-report measures are supplemented by physiological measures of stress and well-being. The inclusion of physiological measures, such as heart rate variability (HRV), may provide a window into the biological origins and consequences of maternal perceptions of their experiences, including the challenges of raising a child with developmental challenges. The proposed project, therefore, involved the collection of multimodal assessment data from mothers and their school-aged children with FXS. Further, given the importance of understanding how mothers of youth with FXS are faring in their everyday environment, the present study collected all data using telehealth procedures and wearable technology. Participants were 20 biological mothers and their children with FXS between the ages of 6 and 11 years. We measured maternal mental health and parenting stress through self-report as well as through HRV as a more objective measure of psychological well-being. We also examined the associations between these variables and child characteristics such as externalizing and internalizing behaviors as well as autism symptomatology. We found significant support for an elevated rate of depressive symptoms in the sample of mothers (35%) and some potential indicators for heightened rates of anxiety (15%) when compared to normed samples and rates in the general population. We also found that the mothers presented with an atypical HRV profile akin to those experiencing depression or chronic stress, although limitations of the present measure suggest the need for additional confirmatory research. Further, we found that child externalizing behaviors were the primary correlates of maternal well-being. These findings contribute to the growing body of literature regarding the unique challenges faced by these mother-child dyads and supports the importance of increasing the availability of services available to these mothers, not only for meeting the needs of their children's development and behavior, but in supporting their own well-being as well.

## Introduction

Considerable research has shown the importance of parenting in supporting typically developing (TD) children's development across multiple domains, including academic achievement and social emotional development (1, 2). Parenting also contributes to the development of children with neurodevelopmental disabilities, such as those with autism spectrum disorder [ASD; (3)], Down syndrome [DS; (4)], or fragile X syndrome [FXS; (5–7)]. However, parents of children with neurodevelopmental disabilities face challenges to parenting, such as increased effort to help their child achieve daily tasks as well as pessimism regarding their child's future compared to parents of TD children (8, 9). Moreover, these parents are often engaged in high levels of advocacy on behalf of their children from birth [or receipt of diagnosis; (10)] and persists through adulthood as they advocate for job placements and more (11). These parents often shoulder additional financial demands surrounding the cost of specialized therapies and health care for their children (12). Parents of children with neurodevelopmental disabilities may also be required to deal with child challenging behaviors, such as aggression and hyperarousal (13, 14), limitations in child adaptive functioning (15–17), as well as social (18, 19) and academic challenges (20, 21), all of which can further contribute to higher caregiver burden and stress. At the same time, however, there are between-syndrome differences in how parents fare; for example, parents of children with FXS often experiencing higher rates of parenting stress and greater impacts to their mental health and well-being relative to parents whose children have Down syndrome [e.g., (8, 22)]. In the case of biological mothers of children with FXS, they are carriers of the genetic alteration that is the source of their children's FXS and a subset may themselves be at an increased risk for anxiety and depression or more subtle symptoms of emotionality such as negative affect in part because of carrying the alteration although genetic factors play a moderating role as well (23–26). Thus, biological mothers of children with FXS are at risk for poorer mental health and stress, which could affect their parenting, by virtue of both their own genetic vulnerabilities and their children's characteristics. These mother-child dyads could benefit from increased support and services to help achieve optimal outcomes for the entire family system. The study reported here focused on understanding the sources of stress and poor psychological well-being in biological mothers of children with FXS to inform the development and delivery of support and services.

### Genetics of Fragile X Syndrome

FXS is the leading inherited cause of intellectual disability and the leading single-gene cause of autism spectrum disorder [ASD; (27, 28)]. FXS is one of several conditions resulting from repetitions of the trinucleotide sequence, CGG, of the fragile X mental retardation 1 (*FMR1*) gene (29). The length of the CGG repeat expansion can ultimately impact neuronal functioning by impacting production of the Fragile X Mental Retardation Protein (FMRP). Expansions between 55 and 200 are considered *premutations*, which occur in 1 in 151 females and is somewhat less common in males (30). Individuals with expansions of 200 or more have the *full mutation* and are most likely to display the behavioral and physical features of FXS (31). Males with the full mutation are more affected than females, on average, because females benefit from the protective effects of a second unaffected X chromosome (32).

Although premutation carriers do not typically have an intellectual disability or display the challenging behaviors associated with FXS, they do have their own unique phenotypic features. Female carriers of the *FMR1* premutation, for example, are more likely to experience mental health related issues such as anxiety and depression (33), as well as physical health complications such as early onset of menopause, infertility, and irregular menstrual cycles reflecting fragile X-associated primary ovarian insufficiency [FXPOI; (34, 35)]. Thus, these women present with a complex physical and emotional phenotypic profile that warrants treatment and support, especially when considering the added impacts of parenting a child with special needs.

### Psychological Well-Being and Parenting Stress in Biological Mothers of Children With Fragile X Syndrome

Individual mental health affects the quality of interactions within a family (36). Biological mothers of individuals with FXS themselves have either the *FMR1* full mutation or, more commonly, the *FMR1* premutation. Thus, a subset of these mothers are at increased risk for mental health concerns, such as depression (37) and anxiety (38, 39), which can affect their parenting. In addition, biological mothers of youth with FXS are more pessimistic about the youth's future, perceive less reciprocated closeness in the parent-offspring relationship, and display more symptoms of depression (8). Mothers of children with FXS have also self-reported lower quality of life (40). Importantly, there is variability among mothers with the *FMR1* premutation in symptom presentation, which may be attributed in part to individual differences in genetic susceptibility and differences in their responses to the stress of raising a child with a disability (25, 41). Moreover, there are biological and social determinants that might explain at least some of the variability in symptom expression including cognitive abilities and life stressors (24, 25, 42). Thus, understanding the source(s) of lowered maternal well-being beyond genetic susceptibility is important for determining optimal paths toward treatment (40).

Children with FXS often display challenging behaviors, such as those consistent with a diagnosis of ASD (43–45), restricted and repetitive behaviors (46, 47), self-injurious behaviors (48), as well as increased rates of anxiety (49, 50). These co-occurring problems impact levels of parenting stress and parental well-being over time (5, 8, 13, 22, 26). Further, high levels of parenting stress may exist throughout the course of the child's development, starting at the time of diagnosis and continuing well into adolescence and adulthood (51). This heightened and prolonged experience of stress can have lasting impacts on the overall health of parents (52, 53). *FMR1* premutation carriers are at an increased risk for developing additional physical health issues, including thyroid disease, hypertension, seizures, peripheral neuropathy, and fibromyalgia (54), which may also contribute to parental stress. Thus, it is important to understand the extent, sources, and consequences of stress experienced by parents to determine ways to better promote health and well-being.

### Measuring Parenting Stress in Mothers of Youth With FXS

Although the vast majority of studies characterizing parenting stress in studies of FXS has involved self-report measures (13, 55), self-report is non-objective and more likely to lead to spurious relationships compared to more objective measures of stress, such as physiological indicators (56). Therefore, in recent years there has been increasing research exploring whether more objective indices of stress, such as physiological markers like cortisol (57, 58), electrodermal activity [EDA; (59)], and heart rate measurements (60, 61) can help us better understand the health and well-being of parents of youth with neurodevelopmental disabilities (62, 63). Measures of the functioning of the autonomic nervous system (ANS) have often been targeted due to its impact on overall health and its role in support of emotional regulation (64) and stress management (65). ANS health and functioning is often indexed by the measurement of heart rate variability (HRV). As opposed to heart rate, which is the continuous beat of the heart, HRV is the time in between heart beats. HRV can be considered under a baseline, or non-stressful at-rest, condition, as well as under a stressful situation for an index of stress reactivity. High HRV at rest reflects overall positive health and optimal emotional regulation (66, 67), and has been associated with increased responsiveness and adaptation to the environment (68). In otherwise healthy individuals, a low HRV at rest could be an indicator of depression (66, 69, 70) or chronic stress (71, 72). Further, the default physiological stress response is characterized by an increase in heart rate, and thus a decrease in HRV, after being exposed to a stressor before ultimately returning to baseline levels (73). Importantly, maladaptive stress responses have been linked to depression (69) and anxiety (74).

There is limited evidence on how HRV and parenting stress are related in parents of individuals with neurodevelopmental disabilities. In a recent study, however, Factor et al. (60) found preliminary support for an atypical stress response as measured by HRV during interactions between mothers and their children with ASD. Specifically, Factor et al. found a positive association between child ASD symptoms and mothers change in HRV, such that a higher rate of symptoms of ASD was related to a larger increase in HRV from baseline. In terms of *FMR1* premutation carriers, a study by Klusek et al. (75) quantified respiratory sinus arrhythmia, an estimate of vagal tone and a metric comparable to HRV, and found support for ANS dysregulation through the observation of reduced vagal tone when compared to study controls. Identifying and treating atypical ANS functioning is especially critical given its relationship to long term health outcomes and therefore should be explored further in this already vulnerable population of *FMR1* carriers. For instance, more research is needed to understand how ANS dysregulation in premutation carriers might be impacted due to parenting stress in particular as well as across other measures of ANS processes which might yield different insights into this complex system. It is also of importance to understand how these mechanisms work in relation to more real-world stressors and interactions such as those between the mother and their child.

### Current Study

Although previous research supports the notion that characteristics of both the child and the mother impact maternal psychological well-being and parenting stress in mothers of children with FXS, more work is needed in which self-report measures are supplemented by physiological measures of stress and well-being. The inclusion of physiological measures, such as the HRV measures used in this study, may provide a window into the biological origins and consequences of maternal perceptions of their experiences, including the challenges of raising a child with developmental delays. The present study used multimodal assessment data, including physiological measures, from mothers and their school-aged children with FXS to further understand maternal mental health, stress, and well-being as they relate to child characteristics. We focused on school-aged children given evidence that parenting stress is highest when children with disabilities are between the ages of roughly 6–11 or 12 years (76, 77). Further, given the importance of understanding how mothers of youth with FXS are faring in their everyday environment, we collected data through online questionnaires, wearable physiological wristbands, interviews with the mother as well as direct assessment of maternal cognitive abilities, and real-time observations of mother-child interactions in the home by means of distance teleconferencing. This form of data collection allowed us to gain more naturalistic and ultimately more generalizable data as well as eased the burden on the families with regards to needing to travel for participation. Two primary hypotheses were addressed:

1. Given their genetic status as carriers of the *FMR1* premutation or full mutation (26), biological mothers of children with FXS, on average, were expected to display elevated rates of mental health symptoms, especially depression and anxiety (39), and parenting stress (41) relative to the general population. We tested this hypothesis using not only self-report measures of well-being but also objective physiological measurement (HRV), making it possible to determine the correspondence between maternal perceptions and physiological indices. We also examined variability in such symptoms among the mothers.
2. Increased maternal mental health symptoms, higher maternal-reported parenting stress (8), and atypical ANS regulation, as measured by HRV (60, 75), were expected to be associated with increased child challenging behavior and symptoms of ASD.

## Materials and Methods

### Participants

Twenty-two mother-child dyads were recruited to participate in a larger study profiling the characteristics of mothers and children with FXS and how these characteristics impact the mother-child interaction. Of the 22 dyads enrolled, 2 discontinued prior to starting data collection and one discontinued after completing only the study questionnaires detailed below. All mothers provided electronic informed consent on behalf of themselves as participants and for their participating child prior to beginning data collection. Families were recruited from around the United States and from Canada through community listservs, university research volunteer registries, and existing lab databases or previous participants. Child participants had a confirmed diagnosis of the full mutation FXS, documented through diagnostic reports shared by parents, and were between 6 and 11 years of age. Both male and female children were recruited to participate (16 males and 4 females) along with their biological mothers. The sample was racially diverse with child participants distributed as follows: 55% White, Non-Hispanic/Latinx, 5% American Indian/Alaskan Native, 5% Black or African American, 15% Hispanic/Latinx, and 20% were multicultural (with 3 of the 4 multicultural families identifying as both Black/African American and White and 1 identifying as American Indian/Alaskan Native and White). Additional eligibility criteria for the child participants were that the child lived at home with the biological mother and that English was the primary language used in the home.

Biological mothers were recruited due to their genetic susceptibility to mental health challenges and increased rates of parenting stress. The participating mothers self-reported on their *FMR1* carrier status, with two mothers having the full mutation, 16 had the premutation, and two never received testing and, therefore, did not know their carrier status. Mothers ranged in age from 28 to 47 years and in IQ from 81 to 131 on the General Ability Measure for Adults [GAMA; (78)]. Participating mothers were 60% White, Non-Hispanic/Latinx, 5% Black or African American, 15% were Hispanic/Latinx, and 20% were multicultural (with 50% indicating both American Indian/Alaskan Native and White and the other 50% indicating both Black/African American and White). Household incomes for the participating families ranged from between 30,001 and 35,000 annually to more than 300,000 annually. For a complete summary of participant characteristics and household information (see Table 1). Overall, the current study sample reflects a largely educated and well-resourced sample of mothers, which is a consistent limitation reported in other studies of this kind in this population [e.g., (5)]; however, the present sample is more culturally diverse than is typical.

**Table 1:** Study participant and household demographics.

	***N***	***%***
**Child participants (6–11 years old;** ***M*** **=** **8.80**, ***SD*** **=** **1.77)**		
Female	4	20
Male	16	80
American Indian/Alaskan Native	1	5
Black or African American	1	5
Hispanic/Latinx	3	15
White, Not Hispanic/Latinx	11	55
More than one race/ethnicity^*^	4	20
**Mother participants (28–47 years old;** ***M*** **=** **40.35**, ***SD*** **=** **5.27)**		
American Indian/Alaskan Native	0	0
Black or African American	1	5
Hispanic/Latinx	3	15
White, Not Hispanic/Latinx	12	60
More than one race/ethnicity^*^	4	20
**Household information**		
**Household income**		
< $50,000	1	5
$50,000–$150,000	9	45
> $150,000	9	45
Preferred not to answer	1	5
**Caregiver status**		
Primary caregiver (mother)	20	100
Two parent/caregiver household	16	80
One parent/caregiver household	4	20

* *75% of the child participants that selected more than once race families endorsed both Black/African American and White, with the remaining 25% indicating American Indian/Alaskan Native and White. 50% of mothers reporting more than one race selected Black/African American and White and the other half selected American Indian/Alaskan Native and White*.

### Procedures

Families were asked to use their own technology for video teleconferencing (i.e., personal tablets or computer). However, in cases in which a family did not have access to technology of their own, we provided a device equipped with video teleconferencing software (e.g., Skype for Business^TM^) and the other applications required to complete the assessments from a distance. Most of the mothers elected to use their own technology, with only two mothers needing technology to be provided. All mothers were provided an Empatica E4 physio-wristband to assess physiological data of the mother in real time. In addition to the physio-wristbands, a wordless picture book and standardized assessment materials were provided. Upon receiving the materials, study staff conducted a technology training session to orient the mother to the technology to be used and to set-up the home environment for optimal data collection. Data were collected by the first author (LB) through a combination of mother-completed online questionnaires, mother interviews, remotely conducted direct assessment of maternal cognitive ability, physiological data, and direct observations of mother-child interactions. Data collection occurred across several distance sessions. All technology applications were HIPAA compliant and, when provided, all computers were encrypted to the specification of the UC Davis Health System.

### Child Measure

In order to provide a comprehensive view of the child factors that were hypothesized to impact maternal well-being and parenting stress, the below measures were collected (Table 2). Measures of child language ability were also derived from the mother-child narratives; however, those measures are not included in this report.

**Table 2:** Means and standard deviations for child and maternal measures.

	***M***	***SD***
**Child measures**		
**Vineland-3**		
Adaptive behavior composite score	67.11	18.66
**Childhood autism rating scale (raw score)**		
Autism symptom severity	28.74	6.17
**Child behavior checklist for ages 6–18 (T-scores)**		
Internalizing behaviors	57.10	8.012
Externalizing behaviors	53.00	9.42
Total behaviors	59.85	9.31
**Maternal measures**		
**General ability measure for adults**		
GAMA IQ score	108.26	12.06
**Symptom checklist 90-revised (T-scores)**		
Anxiety	50.55	10.70
Depression	57.15	9.47
Global severity index	54.80	12.06
**Parenting stress index, fourth edition (T-scores)**		
Child domain	62.55	11.88
Parent domain	53.20	9.26
Total parenting stress	58.20	10.68
**Heart rate variability (millisecond)**		
Baseline HRV	50.09	16.04
Interaction HRV	67.37	22.34
Change in HRV	17.17	15.15

#### Vineland-3

The Vineland-3 (79) was used to assess child adaptive functioning. The Vineland-3 provides scores for specific adaptive behavior domains (i.e., Communication, Daily Living Skills, and Socialization, as well as an overall adaptive behavior composite). The Vineland-3 meets American Association on Intellectual and Developmental Disabilities and DSM-5 requirements for identifying impairments in adaptive behavior as one key component in identifying an intellectual disability. The Vineland-3 was administered as an interview with the mother *via* distance video teleconferencing. For the purposes of the current study, we used the adaptive behavior composite standard score as a proxy for child developmental level, with scores in the present sample ranging from 40 to 127 (*M* = 67.11, *SD* = 18.66). Higher scores reflect more advanced developmental levels. One mother-child dyad discontinued with only partial data collection; therefore, the Vineland-3 was only collected on 19 of the youth participants.

#### Childhood Autism Rating Scale, Second Edition (CARS-2)

Child symptoms of ASD were also assessed using the CARS-2 (80), which is a measure used to identify children with autism and determine symptom severity through quantifiable ratings based on direct observation. The CARS-2 format lends itself readily to use *via* telehealth. The CARS-2 was coded by trained research staff from the recorded mother-child interaction in which the mother and child told a wordless picture book together. Child total scores on the CARS-2 were used as an indicator of symptom severity (with scores between 15 and 29 reflecting minimal-to-no symptoms of ASD, scores between 30 and 36.5 reflecting mild-to-moderate symptoms of ASD, and scores of 37 and higher reflecting severe symptoms of ASD). Scores in the present sample of youth ranged between 16 and 42 (*M* = 28.74, *SD* = 6.17). One mother-child dyad discontinued with only partial data collection; therefore, the CARS-2 was only scored for 19 participants.

#### Child Behavior Checklist for Ages 6–18 (CBCL:6-18)

The CBCL (81) is a parent-report measure of child challenging behaviors and was used to assess child challenging behaviors. The measure creates a composite T-score of both externalizing and internalizing behaviors as well as an overall total challenging behavior T-score. The CBCL was collected on all 20 of the youth participants with scores ranging between 39 and 75 for internalizing (*M* = 57.10, *SD* = 8.01), 34 and 69 for externalizing (*M* = 53, *SD* = 9.42) and between 41 and 74 for total behaviors (*M* = 59.85, *SD* = 9.31).

### Maternal Measures

In order to provide a comprehensive view of maternal factors we collected information regarding mental health status as well as stress (self-reported and physiological, see Table 2), in addition to the more descriptive characteristics such as *FMR1* carrier status and cognitive ability described previously:

#### General Ability Measure for Adults (GAMA; Naglieri and Bardos, 1997)

The GAMA (78) is a 66-item, self-administered timed test (e.g., 25 min) that assesses general cognitive ability of individuals 18 years and older and has been redesigned for implementation *via* telepractice. For this study the mother and research staff (LB) connected *via* video teleconferencing with LB proctoring the administration in real-time. The GAMA was collected on 19 mothers.

#### Symptom Checklist 90-Revised (SCL-90-R)

The SCL-90-R (82) is a 90-item informant report of current psychological problems and symptoms of psychopathology. For each subscale as well as the Global Severity Index (GSI), scores are reported based on a T score distribution with a mean of 50 and standard deviation of 10. T-scores that are ≥63 are considered to be in the clinically significant range (82). In light of the expected phenotype of premutation carriers, we used scores only from the anxiety and depression subscales. The SCL-90-R was adapted to an online questionnaire format and collected for all 20 of the participating mothers.

#### Parenting Stress Index, Fourth Edition (PSI-4)

The PSI-4 (83) is a 120-item informant report questionnaire to be completed by the mother. It contains three major domains of stress: child characteristics, parent characteristics, and situational/demographic life stress. Percentile scores from the PSI-4 are commonly used to interpret clinical status of the parent informant; however, T-scores are also provided based on a normal distribution with a mean of 50 and standard deviation of 10 and are used in subsequent analyses. The PSI-4 was adapted to an online questionnaire format and was collected for all 20 of the participating mothers.

#### Physiological Markers of Stress

Empatica E4 wristbands were used to collect physiological data as a marker of parenting stress in real time. The E4 wristband is a wearable research device designed to collect reliable metrics of stress and health physiology through heart rate measurements and electrodermal activity and is comparable to more clinical based measures including ECG monitors (84, 85). The E4 wristband is equipped with PPG sensors that measure the Blood Volume Pulse (BVP), from which heart rate variability can be derived. The PPG data allows derivation of inter-beat interval (IBI) and thus, HRV. The Empatica E4's algorithm automatically detects abnormal or anomalous heartbeats, often created by motion artifacts, and removes them prior to creating the IBI file, thereby leaving only accurate heartbeats. IBI data files were further inspected visually for any remaining anomalies in the data caused by motion artifacts. From the extracted IBI data, we computed the standard deviation of the IBI of normal sinus beats (SDNN) as our measure of HRV (86). Mothers wore the physio-wristband for 5-min during a baseline, or non-stress inducing, activity (i.e., watching a video of waves crashing on the computer), as well as during the shared telling of a wordless picture book with their child. One mother did not complete either the mother-child interactions or collect physiological data and thus is considered missing for all of the physiological data analyses. Further, one mother encountered technology issues with the wristband and was thus missing data for both contexts, and two other mothers inadvertently turned the watch off after the baseline context and resulting in missing data for the interaction context. Thus, we had useable baseline physiological data for 18 mothers and complete data (i.e., baseline and dyadic interaction with the child) for 16 mothers.

### Data Analysis Plan

To address our first hypothesis, we provided descriptive summaries of symptom severity and stress profiles for the present sample of mothers and computed a series of one-sample *t*-tests using mean T-scores for the study participants against the normative samples for the measures of interest. To address our second hypothesis, we conducted a series of Pearson correlations to examine potential associations between the mother and child variables of interest. Multiple linear regressions were also computed to assess the combined impacts of child characteristics on factors of maternal well-being. Shapiro-Wilk tests of normality were conducted for the primary variables of interest. No tests yielded significant results and thus, the assumptions of normality were met and parametric tests were used in all analyses. To address the potential for Type 1 error due to multiple statistical tests, we conducted Benjamini-Hochberg False Discovery Rate correction. We present both the uncorrected significance levels and flagged significant findings that remained after correction. Analyses were conducted using SPSS statistics software.

## Results

### Profile of Mothers Mental Health and Parenting Stress

**Figure 1:**
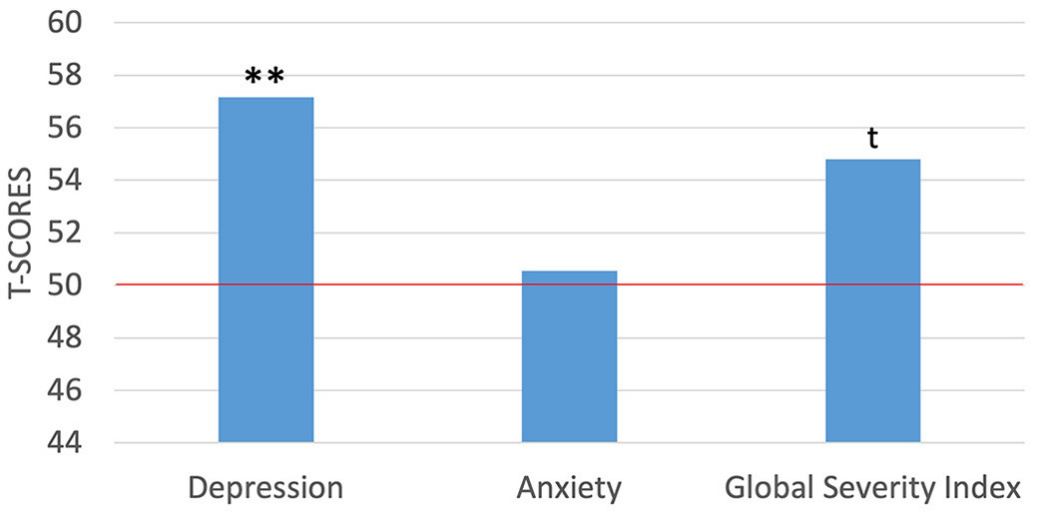
One-sample *t*-tests between maternal self-report of mental health symptoms against the normed sample of the SCL-90-R. **p < 0.01*; t p* = marginally significant. Red horizontal line indicates the normed sample mean of 50.

On average, the mothers in the present sample achieved scores above the means for the general population across a variety of indices of mental health and parenting stress. On the SCL-90-R, the mothers had T-scores between 34 and 71 (*M* = 57.15*, SD* = 9.47) on the depression subscale, scores between 37 and 68 (*M* = 50.55, *SD* = 10.70) on the anxiety subscale, and scores between 37 and 68 (*M* = 54.80, *SD* = 10.55) on the Global Severity Index (GSI). Further, 35% of the mothers reported depressive symptoms consistent with a “case” (i.e., a T-score ≥63 reflecting symptom expression consistent with a clinical diagnosis), which is a notably higher percentage than the national average, which ranges between 10.1% in women between the ages of 20–39 years to 11.5% in women between the ages of 40–59 years (87). The mothers in the present sample also included a higher rate of anxiety “cases,” with a rate of 15% compared to 3.4% for generalized anxiety disorder in the general population of women (88). We further explored how this sample of mothers compared to the normative samples used to develop the measures using *t*-tests and a comparison value of 50 as the hypothesized mean based on a normal distribution. The present sample of mothers reported significantly higher levels of depression [*t*_(19)_ = 3.38, *p* < 0.01], but not significantly higher levels of anxiety. The GSI for the mothers was marginally higher than that of the norming sample [*t*_(19)_ = 2.04, *p* = 0.056; Figure 1]. Moreover, there was some comorbidity of symptom expression with 15% of the mothers reporting symptoms consistent with “caseness” for both depression and anxiety. Lastly, in an exploratory analysis examining how social determinants might relate to the various features of maternal well-being, we found a significant and negative correlation between maternal IQ and depression scores [*r*_(19)_ = −0.471, *p* < 0.05].

**Figure 2:**
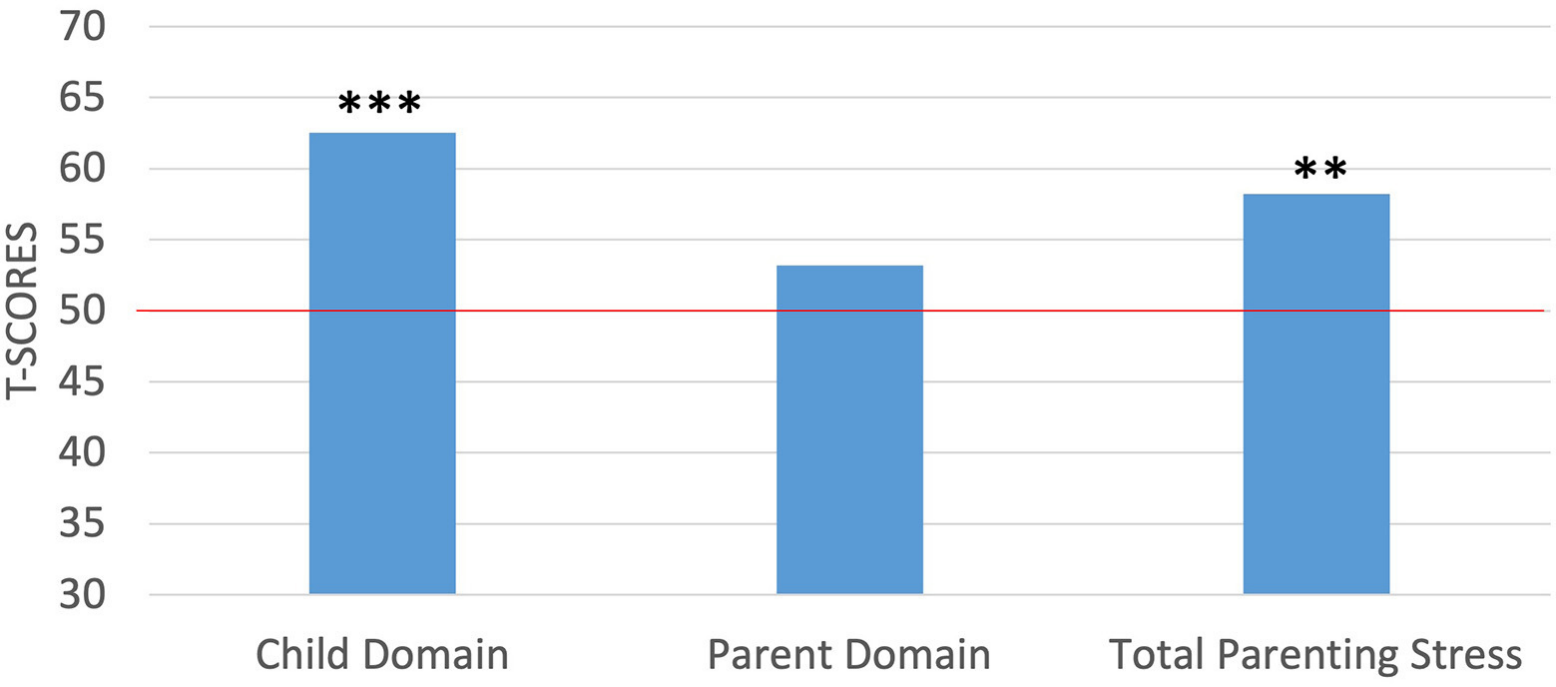
One-sample *t*-tests between maternal self-report of parenting stress against the normed sample of the PSI-4. ***p < 0.001*;* **p < 0.01. Red horizontal line indicates the normed sample mean of 50.

With regards to parenting stress, the mothers in the current study achieved T-scores between 38 and 91 (*M* = 62.55, *SD* = 11.88) on the child domain, between 35 and 75 (*M* = 53.20, *SD* = 9.26) on the parent domain, and scores between 36 and 86 (*M* = 58.20, *SD* = 10.68) for total parenting stress. To compare the study sample of mothers against the normative sample, we again used *t*-tests for the PSI-4 scores following the same procedures outlined above. Results from the PSI-4 indicated that the mothers in the current study reported significantly higher levels of stress in the child domain [*t*_(19)_ = 4.72, *p* < 0.001] and in total stress [*t*_(19)_ = 3.43, *p* < 0.01], but not in the parent domain than did the norming sample (Figure 2). Further, because percentiles are suggested for clinical interpretation on the PSI-4, we examined the distribution of percentile scores in the present sample of mothers with scores below the 85^th^ percentile being within the normal range, scores between the 85^th^ and 89^th^ percentile within the high range and scores in the 90th percentile and higher being considered clinically significant. Percentile scores ranged from 9 to 99% (*M* = 79.05%, *SD* = 23.26) for the child domain, 3 to 99% (*M* = 61.95%, *SD* = 26.43) on the parent domain, and between 3 and 99% (*M* = 72.20%, *SD* = 23.79) for total stress. Notably, the mothers scored within the clinically significant range at a higher than expected rate of 10% for the normative sample in two instances: 30% of the mothers scored within the clinically significant range for the child domain and 20% scored within the clinically significant range for total stress level. For the remaining PSI-4 domains, maternal scores for were more consistent with the expected distributions from the normative sample, with 15% of the mothers scoring within the high range on the child domain, 10% of the mothers scoring within the clinically significant range for the parent domain (all others were within the normal range), and 15% scoring within the high range for total stress.

The final metric of stress was HRV collected during the baseline activity (sitting while watching a video of waves crashing) and during an interaction with their child (sitting while telling a wordless picture book story with their child). There was a significant difference between the two conditions (*t* = −4.241, *p* < 0.01) with mothers in the current study having an HRV between 17.65 and 72.03 ms (*M* = 50.09 ms, *SD* = 16.04) during the baseline activity and an HRV between 30.31 and 109.94 ms (*M* = 67.36 ms, *SD* = 22.34) during the wordless picture book interaction with their child. Change in HRV between the two contexts ranged from a decrease of 15.16 ms to an increase of 48.87 ms, with a mean increase in HRV between the two contexts of 17.17 ms (*SD* = 15.15). On average, the profile of stress reactivity in the mothers in the present study contrasts with that expected for a typical and healthy stress response (73) and more comparable to individuals experiencing depression (89) or chronic stress (90).

Further, Shaffer and Ginsberg (86) suggest that individuals who have an HRV below 50 ms during a resting condition are unhealthier, those with HRV between 50 and 100 ms have compromised health, and those with HRV over 100 ms are healthy. Based on these guidelines, 44% of the mothers had a resting HRV in the unhealthy range (i.e., below 50 ms during the baseline activity), and the remaining 56% had a resting HRV in the compromised health range. In contrast, another study examining the average HRV in the general healthy population suggests an approximate HRV of 50 ms with a standard deviation of 16 (91), which is quite similar to the findings for the present sample of mothers as well.

### Maternal Well-Being as It Relates to Child Characteristics

**Figure 3:**
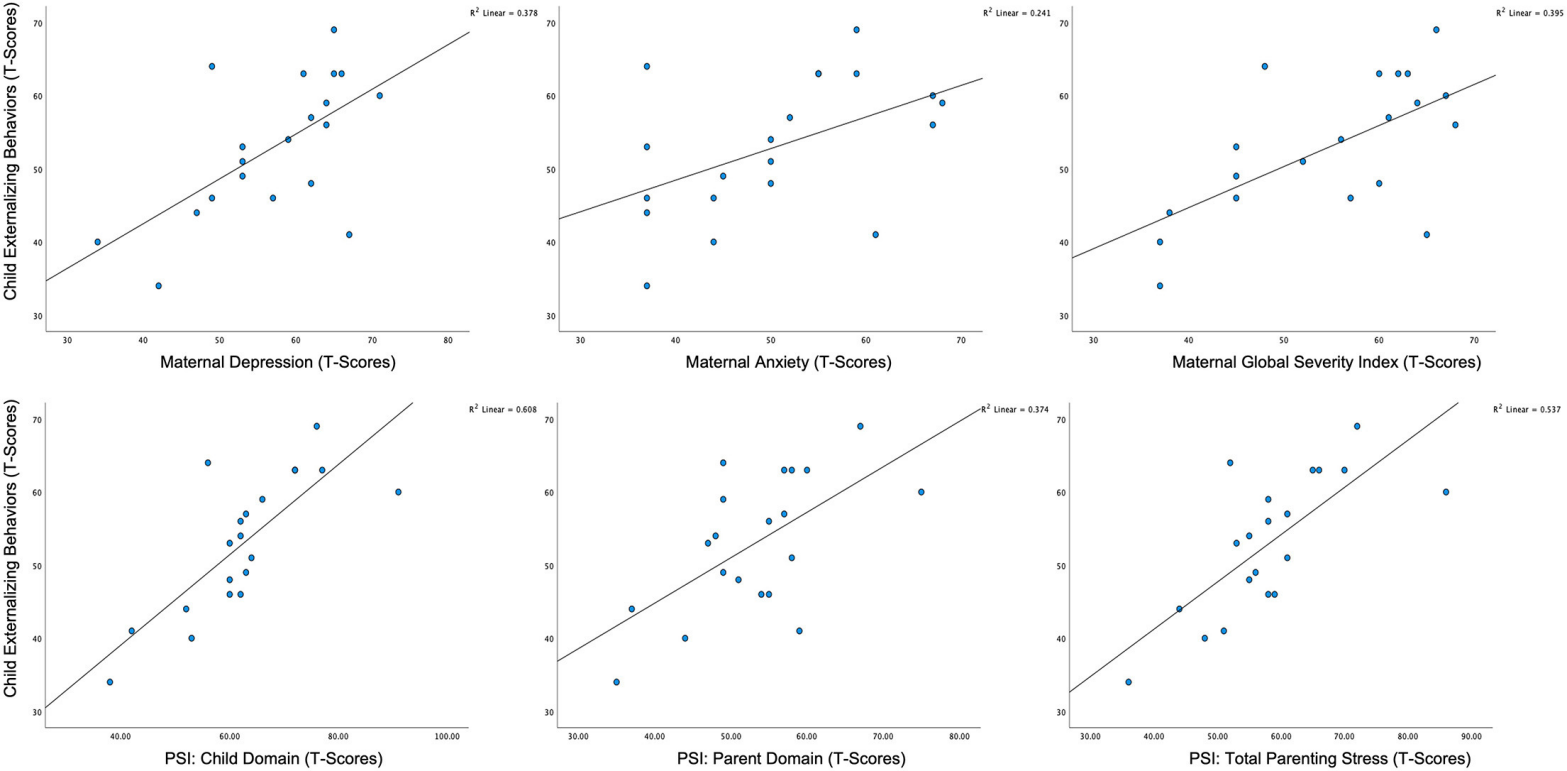
Scatterplots of associations between child externalizing behavior and maternal mental health status and parenting stress.

A series of two-tailed, Pearson correlations were computed to assess relationships between measures of maternal well-being and child characteristics. For self-report measures of maternal mental health and parenting stress, we found that child externalizing behaviors, as measured by the CBCL, were positively correlated with all the maternal variables of interest (see Table 3; Figure 3), and this finding remained significant after FDR correction. Further, we found that child adaptive behavior, as measured by the Vineland-3, was also significantly and negatively correlated with the child domain score on the PSI-4; however, this correlation was no longer significant after the FDR correction. To assess the combined effects of child externalizing behaviors and child adaptive functioning on the child domain scores on the PSI-4, we computed a multiple linear regression. The overall model was significant [*F*_(2, 17)_ = 14.239, *p* < 0.001, *R*^2^ = 0.648], with child externalizing behaviors emerging as the only unique predictor (*t* = 3.89, *p* < 0.01). There were no significant associations between child characteristics and HRV.

**Table 3:** Correlations between maternal measures of mental health and stress and child characteristics.

	**1**	**2**	**3**	**4**	**5**	**6**	**7**	**8**	**9**	**10**	**11**	**12**	**13**	**14**	**15**
**Parenting stress index-4**															
1. Child domain	-														
2. Parent domain	0.771^**^	**-**													
3. Total stress	0.951^**^	0.928^**^	-												
**Symptom checklist 90-revised**															
4. Depression	0.608^**^	0.754^**^	0.718^**^	-											
5. Anxiety	0.534^*^	0.661^**^	0.626^**^	0.799^**^	-										
6. Global severity index	0.574^**^	0.772^**^	0.701^**^	0.941^**^	0.884^**^	-									
**Heart rate variability**															
7. Baseline HRV	−0.067	−0.020	−0.035	0.006	−0.225	−0.070	-								
8. Wordless Picture Book Interaction HRV	−0.092	0.122	0.006	−0.021	−0.244	−0.126	0.735^**^	-							
9. Change in HRV	−0.059	0.209	0.062	−0.081	−0.240	−0.183	−0.005	0.674^**^	-						
**Child characteristics**															
10. Age	0.221	0.115	0.212	0.040	0.154	0.091	0.235	−0.159	−0.411	-					
11. Adaptive functioning	−0.554^*^	−0.122	−0.371	0.053	−0.054	−0.031	0.220	0.254	0.217	−0.085	-				
12. Internalizing behaviors	0.082	0.054	0.054	0.199	0.053	0.244	−0.194	−0.239	−0.198	0.080	−0.094	-			
13. Externalizing behaviors	0.780^**^	0.611^**^	0.733^**^	0.615^**^	0.491^*^	0.629^**^	0.058	−0.125	−0.294	0.253	−0.415	0.460	-		
14. Total behaviors	0.620^**^	0.411	0.538^*^	0.503^*^	0.359	0.515^*^	−0.073	−0.189	−0.278	0.133	−0.417	0.681^**^	0.921^**^	-	
15. ASD symptomatology	0.388	0.014	0.229	−0.207	−0.046	−0.135	0.055	−0.240	−0.231	0.120	–0.629^**^	0.050	0.224	0.222	-

** *Correlation is significant at the 0.01 level (2-tailed)*.

* *Correlation is significant at the 0.05 level (2-tailed)*.

*The bolded correlations remained significant after accounting for multiple comparisons using the Benjamin-Hochberg procedure to detect the false discovery rate*.

## Discussion

The present study was designed to build on previous research by exploring the phenotypic characteristics of biological mothers of children with FXS and determine how child characteristics might contribute to aspects of maternal well-being. All data were collected in the family home through various forms of technology, from video teleconferencing to digital wearables, through virtual interactions. Although preliminary because of the relatively small sample size, the findings from the current study are consistent with and extend previous findings regarding the compromised psychological well-being of at least a subset of biological mothers of children with FXS [e.g., (23, 39)], thereby suggesting that remote data collection yields similar findings as does questionnaire-based and in-person studies. This study also provided novel findings as regards to physiological measurement of stress suggesting through wearable technology to measure heart rate, demonstrating the feasibility of distance data collection though wearable technology. More generally, the findings indicate elevated rates of depression and parenting stress, as measured through both self-report and a physiological measure (HRV), in biological mothers of children with FXS as well as a relationship between child externalizing behavior and different facets of maternal psychological well-being. These findings highlight the need to address the mental health and stress facing mothers as well-target child challenging behaviors to help achieve optimal family functioning.

### Profiles of Maternal Well-Being

Consistent with previous findings in the literature, the mothers in the present study had elevated rates of depression compared to the general population (33, 37). In contrast to previous studies showing higher rates of anxiety in *FMR1* carriers (38, 39), the mothers in the present study were not significantly higher in anxiety when compared to the normative sample; however, the difference relative to the general population just failed to reach statistical significance and could be attributed to a lack of power given our sample size. Thus, the risk for anxiety disorders in these women should not be ignored. Given the importance of mental health to overall physical health and well-being and to the quality of parenting, support should be made available routinely to these mothers in order to help them deal with, or even prevent, these mental health challenges. Although in the larger literature, symptoms of depression and anxiety have been shown to positively respond to pharmaceutical treatments and interventions such as seeing a counselor/therapist in the general population, the efficacy of these treatments has not been fully explored in this specific population. It is also possible that increased support (92), counseling (93), and respite services (94) provided to parents, and particularly at critical points in the development of the individual with FXS (e.g., the transition to school, the onset of puberty), could even prevent or forestall the mental health challenges experienced by these mothers. Future research should consider how we can capture the current use of such services as well as their efficacy given the unique phenotypic characteristics and experiences of biological mothers of children with FXS.

In addition to individual mental health and well-being, because of the nature of their maternal caregiving role for children with special needs, we also explored their profiles of parenting stress. The mothers in the present study had significantly higher feelings of parenting stress, especially with regards to child-associated stressors, when compared to a normative sample of parents. This finding is also consistent with the literature in that mothers of children with neurodevelopmental disabilities such as FXS are at elevated risk of experiencing parenting stress when compared to those of more typically developing children (22, 95). Given the fact that the caregiver role for parents extends well-beyond what is typical given the high level of dependence individuals with FXS require, even into adulthood (96), these mothers are also at a risk for experiencing chronic stress that ultimately could negatively impact their overall physical as well as mental health (97). Importantly, there is evidence to suggest the importance of increasing self-care practices in such chronically stressed caregivers in supporting stress management (98, 99). Indeed, a recent study has documented the benefits of a mindfulness training intervention for *FMR1* premutation carriers (100). This potential area for intervention is particularly important given recent findings by Wheeler et al. (101), suggesting that mindfulness played a protective roll across multiple metrics of maternal well-being, including anxiety, depression, stress, and overall health. Perhaps mindfulness could be thought of as not only a way to provide coping strategies for parents experiencing elevated levels of stress and poor well-being, but also a preventive strategy to avoid poor psychological well-being in these women. Moreover, continued implementation of behavioral interventions that target child characteristics such as those found to relate to maternal well-being and parenting stress in the present sample (e.g., externalizing behaviors like irritability, aggression, etc.) are paramount to the meeting the needs of the whole family especially when parents are included in their child's treatment plan [e.g., through parent mediated interventions; (102, 103)].

In addition to self-report measures of parenting stress and well-being, a novel aspect of the present study, we also explored the role HRV plays in understanding maternal stress and overall health and well-being in this population of mothers. We considered maternal HRV across two contexts, first during a seated resting state/non-stressful activity and then during a seated interaction with their child (e.g., telling a story together) that followed immediately. From a review of the larger literature, it is thought that a higher resting HRV is indicative of physiological resilience to stressors and that further, in healthy individuals, when they are faced with a stressor, a typical profile is for HRV to decrease in response (72), although there is considerable individual variability and some inconsistency across studies in this regard (69). In the present study, mothers had relatively low HRV during the resting condition which then increased on average during the mother-child interaction. Notably, this HRV profile is consistent with those seen in individuals with depression (104), as well as in those who are experiencing chronic stress (71, 105). Thus, our findings suggest a convergence of self-report and HRV in mothers who carry either full mutation or premutation at least in regards to depression. Given the relatively short time period of measurement and limited contexts of measurement in this study (i.e., 5 min during the baseline context and an average of 7 min during the mother-child interactions), it is not possible to discern whether the physiological findings are more indicative of traits or transient, situationally determined, states of stress. This should be explored further through the use of longer term HRV measurement across multiple contexts, as well as through other physiological indices of the ANS such as EDA and vagal tone. Moreover, the nature of such parent-child interactions (e.g., the presence of child challenging behaviors, preferred vs. non-preferred tasks, etc.) should be explored further to identify why they might elicit a greater stress response from these parents and how supports can most effectively be implemented. Moreover, although we removed motion artifacts from the data and both the baseline and dyadic interaction contexts entailed the mother being seated, it is possible that more subtle physical movements occurred in the two contexts. Future research should include additional control for movement as well as a similarly assessed and appropriately matched comparison group of mothers and their typically developing children to ensure that the differences in context we observed are, in fact, non-normative.

Although the HRV profiles observed in this study are consistent with what we found through parent self-report, exploratory analyses indicated no relationship between HRV and self-report indices of stress and well-being for the mothers in the present study (Table 3). This lack of relationship is, however, consistent with other studies of a similar nature looking at HRV in premutation carriers (75) and mothers of children with ASD (60) providing reason to believe that these two metrics capture different components of the individual's functioning and should, therefore, be further explored in tandem to gain a complete picture. Further, as with the findings for self-report measures of stress and well-being, mindfulness interventions impact HRV in positive ways as well (106, 107) and thus, could prove even more beneficial in populations such as mothers of children with FXS who display concerning profiles on both metrics of stress. Further, additional characteristics of the mother should be considered when implementing treatments. For instance, our finding regarding the relationship between depression and IQ might also suggest the need for different approaches to intervention among mothers of children with FXS.

### Child Determinants of Maternal Well-Being

Lastly, we found considerable support for the role that the behavioral profile of the child with FXS, especially his or her level of externalizing behavior, plays in various aspects of maternal well-being; again, consistent with previous findings. Though limited by a small sample size and measurement at a single time point, which leaves the causal direction of the relationship unclear, the association between the two is consistent with the basic principles of the transactional model of development. In particular, the relationships are indicative that the child and their environment are bidirectionally interconnected (108). Thus, in addition to supporting the mother as the individual, it is important to continue building upon prior work aimed at optimizing child outcomes, which could ultimately also have an added benefit for the well-being of the mother.

### Limitations

This project is limited by a small sample size and thus many of our conclusions are considered preliminary and in need of further examination in a larger sample. This data set is also limited due to its lack of comprehensive information on genetic affectedness on behalf of the mothers beyond self-report of carrier status and thus future studies should explore more nuanced measures of genetic susceptibility to well-being not able to be examined here. Further, the mothers represented a relatively well-educated and resourced group and thus more work is needed to discern how our findings might generalize to a more under resourced sample. Also, given that data were collected at only one time point, future work would benefit from a more longitudinal approach to determine the bidirectional relationships of the associations we found. At the same time, the present sample of families was racially diverse, which may have been an outcome of our use of fully remote data collection procedures. These procedures may also have increased the representatives of the responses and behaviors of study participants who were able to complete the study in the familiar setting of the family home largely on a schedule that was maximally convenient for them. The inclusion of both self-report and physiological measures of stress was an additional positive and innovative feature of the study.

It is of note that our data collection was partially impacted by the COVID-19 pandemic, which contributed somewhat to recruitment challenges as well as contributing to one mother discontinuing early. Although in some instances, we observed increased availability of families due to being home, it also increased the level of stress and uncertainty per parent report during screening calls and they felt less inclined to add more burden to their own or their child's schedule by participating in a study, even one that did not require leaving home. Further, with regards to the data that were collected, 10 of the mother-child dyads completed the study prior to shelter-in place orders and the remaining 10 dyads completed data collection during the pandemic. We did assess potential differences on the dependent measures of maternal stress and well-being and child challenging behavior and found no differences between the two groups of mother-child dyads. These findings suggest that perhaps the families had adapted to conditions of the pandemic and were no more or less stressed than in the pandemic during pre-pandemic life, although it must be acknowledged that the small sample size and limited statistical power makes any conclusions tentative.

## Conclusions and Future Directions

Overall, the profiles of mental health status and parenting stress are reflective of what is seen in the larger literature as well as the relationship between child challenging behaviors and maternal well-being, which provides continued implications for the roll that supporting child development and behaviors can ultimately have on aspects of maternal well-being. Further, there were unique findings derived from this study including unique physiological profiles of stress and well-being that were separate from more standard measures (e.g., questionnaires) of stress and well-being in *FMR1* carriers. Moreover, a relative strength of this study is the use of multiple formats for measuring maternal well-being, including self-report as well as potentially more objective measures of well-being through the collection of maternal HRV. Another strength of the current study is the completion of the project entirely *via* distance through the use of video teleconferencing and online, electronically completed questionnaires. Further, implications across all of these findings support the need for dual support for both the mother and the child in order to achieve optimal outcomes. Despite the interconnectedness and importance of this relationship, however, there is a relative dearth of research on such combined interventions supporting multiple family members simultaneously.

## Data Availability Statement

The raw data supporting the conclusions of this article will be made available by the authors, without undue reservation.

## Ethics Statement

The studies involving human participants were reviewed and approved by the Institutional Review Board at the University of California, Davis. All mothers provided electronic informed consent on behalf of themselves as participants and for their participating child prior to beginning data collection.

## Author Contributions

The submitted research was part of LB's dissertation research project. LB assisted with the design of the project and collected and analyzed all study data and wrote the corresponding manuscript. DH provided guidance on the statistical analysis plan. LA was the graduate advisor to LB and assisted her with the design of the project and oversaw data collection. All authors reviewed, edited, and approved this manuscript.

## Conflict of Interest

The authors declare that the research was conducted in the absence of any commercial or financial relationships that could be construed as a potential conflict of interest.

## Publisher's Note

All claims expressed in this article are solely those of the authors and do not necessarily represent those of their affiliated organizations, or those of the publisher, the editors and the reviewers. Any product that may be evaluated in this article, or claim that may be made by its manufacturer, is not guaranteed or endorsed by the publisher.
